# Altered circRNAs: a novel potential mechanism for the functions of extracellular vesicles derived from platelet-rich plasma

**DOI:** 10.3389/fbinf.2025.1690932

**Published:** 2026-01-08

**Authors:** Lifeng Niu, Yanli Wang, Yao Gao, Jun Zhang

**Affiliations:** 1 Department of Orthopedics, Nantong Hospital of Traditional Chinese Medicine, Nantong, China; 2 Department of Orthopedics, Nantong Hospital of Nanjing University of Chinese Medicine, Nantong, China; 3 Department of Orthopedics, The Second Hospital of Jilin University, Changchun, China; 4 Department of Orthopaedic Rehabilitation and Pain, The Second Hospital of Jilin University, Changchun, China

**Keywords:** circular RNA, extracellular vesicles, platelet-rich plasma, bioinformatics analysis, potential mechanism

## Abstract

Platelet-rich plasma (PRP) has been widely applied in clinical practice for tissue repair and regeneration. Recent studies have reported that large amounts of extracellular vesicles (EVs) derived from PRP (PRP-EVs) are also involved in the functions of tissue repair and regeneration, except for the secreted growth factors. However, the relevant mechanisms of PRP-EVs remain unknown. In this study, we attempted to reveal the potential circular RNA (circRNA) mechanisms of PRP-EVs using high-throughput RNA sequencing (RNA-seq) technique and bioinformatics analysis. Six healthy donors were enrolled in this study, including three donors for the isolation of PRP-EVs and three donors for the isolation of EVs derived from blood plasma (plasma-EVs). As a result, we confirmed that PRP activation by thrombin could significantly promote the formation and secretion of EVs, particularly those with diameters ranging from 50 to 200 nm. Moreover, 144 circRNAs were altered in PRP-EVs with a fold change ≥ 2.0 and p-value ≤ 0.05. Among these, 89 circRNAs were upregulated, whereas 55 circRNAs were downregulated. Gene Ontology (GO), Kyoto Encyclopedia of Genes and Genomes (KEGG) pathway, and circRNA–miRNA–mRNA interaction network analyses were performed to predict the potential roles of circRNAs in PRP-EVs. GO analysis indicated that these altered circRNAs might be related to the physiological processes of cell genesis and development. The pathways that were most strongly correlated with the biological functions of PRP-EVs were the transforming growth factor β (TGF-β) signaling pathway and HIF-1 signaling pathway. In addition, the expression levels of five selected circRNAs were verified through RT-qPCR. In conclusion, this is the first study to explain a novel potential mechanism of the biological functions of PRP-EVs in terms of the altered circRNAs. Taken together, our findings in this study may lay the groundwork for the clinical application of PRP-EVs and provide possible novel targets for further research.

## 1 Introduction

Platelet-rich plasma (PRP) is a well-established biological derivative of autologous peripheral blood containing a supra-physiological concentration of platelets (Rui et al., 2024). Due to its safety, efficacy, minimal toxicity, and ease of preparation, PRP has been widely used in bone regeneration (Joshi and Duvic, 2024), articular cartilage repair (Wang et al., 2024), wound healing (Mohammadivahedi et al., 2024), tendon and ligament repair (Qiu et al., 2024), central and peripheral nerve repair (Fossati et al., 2024; Sanchez et al., 2024), and osteoarthritis therapy (Wang et al., 2024). As reported in previous studies, these potential functions of PRP in tissue repair and regeneration are attributed to various growth factors that are secreted by the activation of platelets, including platelet-derived growth factor (PDGF), transforming growth factor β (TGF-β), insulin-like growth factor 1 (IGF-1), epithelial growth factor (EGF), basic fibroblast growth factor (bFGF), and vascular endothelial growth factor (VEGF), among others (Lubkowska et al., 2012). These biological agents play a crucial role in promoting tissue repair and regeneration by improving cellular proliferation, migration and differentiation, angiogenesis, collagen synthesis, anti-inflammatory effect, and the synthesis of extracellular matrix (Karaborklu et al., 2024; Nie et al., 2024). However, recent studies have found that, except for these growth factors, PRP also secreted large amounts of extracellular vesicles (EVs) which are involved in the regulation of tissue repair (Torreggiani et al., 2014). EVs are secreted from various types of cells and exist in various types of body fluids, including exosomes (Exos, 20 nm–100 nm in diameter) and microvesicles (MVs, 100 nm–1,000 nm in diameter), which play an important role in cell-to-cell communication as vehicles to transfer the cytosolic proteins, lipids, and RNAs between different cells (Hajialiasgary Najafabadi et al., 2024; Raposo et al., 2024; Tao et al., 2017a).

Torreggiani et al. (2014) isolated exosomes (a specific type of EVs) from PRP using the differential ultracentrifugation method and demonstrated their outstanding functions in the proliferation, migration, and deposition of mineralized matrix in bone marrow stromal cells. Subsequently, Tao et al. (2017b) confirmed that the exosomes derived from PRP (PRP-Exos) exhibit strong ability to stimulate cell proliferation, angiogenesis, and osteogenesis and inhibit cell apoptosis via the Akt/Bad/Bcl-2 signaling pathway for the treatment of osteonecrosis of the femoral head. In addition, Guo et al. (2017) also found that PRP-Exos can promote cell proliferation, migration, angiogenesis and the re-epithelization of chronic cutaneous wounds via activating Yes-associated protein. Tao et al. (2017a) reviewed the important role of platelet-derived EVs in tissue repair and tumor progression.

Nevertheless, the relevant mechanisms of biological functions of PRP-EVs are still unknown. Currently, many studies have suggested that the excellent biological functions of PRP-EVs are mainly attributed to the encapsulated growth factors such as PDGF, TGF-β, bFGF, and VEGF. These results revealed that PRP-EVs contain a higher amount of growth factors than PRP (Torreggiani et al., 2014; Tao et al., 2017b; Guo et al., 2017). With regard to RNA content, an enrichment of microRNAs in platelet-derived exosomes compared to platelets has also been reported (Dempsey et al., 2014). Moreover, circular RNAs (circRNAs) are enriched in platelet-derived EVs, which are selectively released by these EVs (Preusser et al., 2018). circRNAs are a special type of endogenous non-coding RNAs formed by backsplicing events (Liang et al., 2024). Currently, although the main functions of circRNAs largely remain unknown, accumulating evidence suggests that they function as sponges of miRNAs, regulators of splicing and transcriptional events, modifiers of parental gene expression, and templates for protein translation (Legnini et al., 2017). Therefore, we suspected that some circRNAs might be differentially expressed in PRP-EVs and play a crucial role in influencing these functions of tissue repair and regeneration. However, the expression profiles and the potential predictions of biological functions of circRNAs in PRP-EVs are currently unknown.

To this end, high-throughput RNA sequencing (RNA-seq) was performed to identify differential expression profiles of circRNAs between PRP-EVs and plasma-EVs in this study. The potential biological functions of altered circRNAs were predicted through bioinformatics analysis. In addition, the relevant analyses for activating PRP were also explored. The circRNA–miRNA–mRNA interaction network involving five randomly selected circRNAs was also established. Taken together, this study reveals the potential mechanisms of circRNAs in PRP-EVs involved in tissue repair and regeneration.

## 2 Materials and methods

### 2.1 Donors and isolation of PRP or plasma

Six healthy donors were enrolled in this study. Among them, three donors were randomly selected for the isolation of PRP-EVs, whereas the remaining three donors were selected for the isolation of plasma-EVs. All donors were between 18 and 50 years of age and had no history of anemia, blood diseases, severe chronic diseases, and advanced tumors. This study protocol was approved by the Ethical Committee of The Second Hospital of Jilin University. All donors provided written informed consent.

Human PRP was isolated as described previously (Wu et al., 2016; Wu et al., 2017). In brief, 50 mL of peripheral venous blood sample from each donor was collected in the anticoagulant tubes with sodium citrate under sterile conditions. Then the lower layer of erythrocytes were removed after the first centrifugation at 200 × g for 10 min, and the upper layer of platelet-poor plasma was removed after the second centrifugation at 400 × g for 10 min. After this two-step centrifugation, almost 6 mL of plasma remained as PRP. For the isolation of plasma, 20 mL of the peripheral venous blood sample was collected using the same method. Subsequently, the blood sample was centrifuged at 1,500 × g for 15 min for the separation of plasma and hemocytes. The upper layer of plasma was collected for the isolation of plasma-EVs.

### 2.2 PRP activation and EV isolation

To observe the influences of PRP activation on the formation and secretion of EVs, we further compared the concentration of secreted EVs between PRP and activated PRP using nanoparticle tracking analysis (NTA). Based on the previous articles, this study used thrombin as the additional activator for PRP activation (Aatonen et al., 2014). PRP was incubated in 1 U/mL thrombin solution (Sigma-Aldrich) for 30 min at 37 °C. Subsequently, EVs were extracted from PRP, activated PRP, and plasma.

For the isolation of EVs from PRP or activated PRP, the differential ultracentrifugation method was used as described previously (Aatonen et al., 2014). In brief, 1 mL PRP solution was diluted with 5 mL PBS. This diluted PRP solution was first centrifuged at 2,000 × g for 20 min at room temperature to remove platelets. Then the supernatant was transferred into new tubes, and the cell debris was removed through centrifugation at 10,000 × g for 40 min. Finally, the small PRP-EVs were isolated through ultracentrifugation at 160,000 × g for 2 h at 4 °C. After being washed twice, the remaining pellet was resuspended in 100 µL filtered PBS for subsequent experiments. To isolate plasma-EVs, the collected plasma was processed to remove residual cells and cell debris, and the EVs were then isolated using the same differential ultracentrifugation method as for PRP-EV isolation.

### 2.3 Nanoparticle tracking analysis

The concentration and size distribution of total EVs from PRP and activated PRP were determined using NTA using NanoSight NS300 (Malvern, United Kingdom). First, all EV samples were diluted in filtered PBS (1:100) to obtain the optimal concentration for detection (10^6^–10^9^ particles/mL) before being introduced into the instruments. Then the samples were analyzed using NanoSight NS300 equipped with sCMOS camera and Blue405 laser with default settings. For each sample, videos were recorded for a total of 60 s. The results were analyzed using NTA 3.2 software. Finally, the difference in the concentration of secreted EVs between PRP and activated PRP is considered the reflection of the influence of the PRP activator on the secretion of EVs.

### 2.4 Characterization of PRP-EVs and plasma-EVs

The PRP-EVs from activated PRP and plasma-EVs were further collected to elucidate the potential mechanisms underlying the biological functions of PRP-EVs. The PRP-EVs and plasma-EVs were isolated as described above, and their characterizations were determined. First, transmission electron microscopy (TEM, JEM-2100F, Japan) was utilized to observe the micromorphology of EVs. In brief, a drop (10 µL) of EV solution was pipetted onto a formvar-coated copper grid. After standing for 5 min, the excess fluid was removed. Then the sample was negatively stained with 10 µL 3% (w/v) phosphotungstic acid for 5 min. After air-drying under an electric incandescent lamp, TEM detection was performed. Second, the EVs were characterized by Western blotting using the EV surface marker TSG101 (Sigma-Aldrich) and the platelet marker CD41 (Sigma-Aldrich). The EV samples were lysed in RIPA buffer on ice to collect total protein, and the protein concentration was determined using a BCA assay kit (Thermo Fisher Scientific, United States). Western blotting was performed according to the manufacturer’s instructions. Finally, the size distribution of PRP-EVs and plasma-EVs was also examined by NTA, as described above.

### 2.5 RNA extraction, RNA library construction, and RNA sequencing

According to the manufacturer’s protocol, total RNA was extracted from the EVs using the exoRNeasy Serum/Plasma Midi kit (Qiagen, CA, United States). EV suspension was mixed with binding buffer (XBP) at a ratio of 1:1, and the mixture was transferred to the exoEasy spin column. After centrifugation at 500 × g for 1 min, the flow-through was discarded, and 3.5 mL wash buffer (XWP) was added to the spin column. After another centrifugation and discarding of the flow-through, 700 µL QIAzol was added to lyse EVs, and the lysate was collected. Following incubation for 5 min, 90 µL chloroform was added and incubated for 2–3 min. After centrifugation at 12,000 × g for 15 min, the upper aqueous phase was collected and mixed with ethanol. This mixture was transferred to the RNeasy MinElute spin column and was separated through centrifugation. The column was washed once with RWT buffer, followed by twice with RPE buffer and once with RNase-free water. Finally, total RNA was collected for the subsequent experiments.

The concentration and purity of total RNA were determined in each sample by OD260/280 readings using NanoDrop ND-1000 (Thermo Fisher Scientific, Waltham, MA, United States). Due to the differences in RNA production among various EV samples, only RNAs with sufficient yield were subjected to denaturing agarose gel electrophoresis to assess RNA integrity; for samples with extremely low RNA yields below the detection limit, RNA integrity was analyzed through the fragment distribution of the subsequent libraries. Following the manufacturer’s instructions, rRNAs were removed from total RNA using the Ribo-Zero rRNA removal kit (Illumina, United States). The rRNA-depleted RNAs obtained with the TruSeq Stranded Total RNA Library Prep kit (Illumina, United States) were used to construct the RNA libraries. In addition, the quality control and quantification of the libraries were performed in the Bioanalyzer 2100 system (Agilent Technologies, United States).

The RNA-seq was performed by CloudSeq Biotech Inc. (Shanghai, China). The total RNA sequencing libraries were constructed according to the Illumina standard protocol. Depending on the initial RNA quantity, we utilized two library construction strategies: for samples with an input quantity higher than 1 ng, we used the low-input library kit; for samples with an input quantity lower than 1 ng, we adopted the low-input RNA amplification protocol. All library constructions use the quantified RNA before amplification as the initial input quantity. Through *in situ* amplification, clusters were formed. Finally, dual-end sequencing was performed on the Illumina NovaSeq 6000 platform with a read length of 2 × 150 base pairs, completing 150 cycles of sequencing. After quality-control filtering, each sample yielded approximately 40 million high-quality sequences on average, with an average sequencing depth of 40 million reads, ensuring sufficient transcriptome coverage and supporting reliable subsequent analyses.

### 2.6 Bioinformatics analysis

After RNA sequencing, quality control was first performed on the raw FASTQ files using FastQC to assess sequencing quality. Adapter sequences and low-quality bases were removed using Trimmomatic to ensure that the subsequent analyses were based on high-quality reads. Subsequently, the quality-controlled reads were aligned to the reference genome/transcriptome using STAR, and circRNAs were identified using DCC software. All identified circular RNAs were annotated using the circBase database and were analyzed for differential expression using edgeR.

To further explore the biological functions of circRNAs, we conducted Gene Ontology (GO) (http://www.geneontology.org) and Kyoto Encyclopedia of Genes and Genomes (KEGG) pathway analyses (http://www.genome.jp/kegg) on the differentially expressed circRNAs. Additionally, based on the mechanism that circRNAs may act as miRNA molecular sponges to participate in gene regulation, we combined the TargetScan and miRanda databases and used Arraystar miRNA target prediction software to construct a potential interaction network between circRNAs and miRNAs. Finally, Cytoscape was used to draw a regulatory network diagram of five randomly selected differentially expressed circRNA–miRNA–mRNA interactions (http://www.cytoscape.org/download.html).

### 2.7 RT-qPCR validation

To verify the accuracy of the RNA-seq data, RT-qPCR analysis was carried out. The RNA samples used in RT-qPCR analysis were of the same origin as those used in RNA sequencing. Five differentially expressed circRNAs were randomly selected for further verification: chr2:72945232-72960247− (hsa_circ_0009043), chr3:114069121-114070725− (hsa_circ_0005332), chr10:126370176-126370948− (hsa_circ_0000267), chr5:142416761-142437312+ (hsa_circ_0074368), and chr5:76342172-76344097+ (hsa_circ_0008620). Total RNA was reverse-transcribed to synthesis cDNA using random primers with the PrimeScript RT Reagent kit (Takara, Osaka, Japan). RT-qPCR was performed using TB Green® Premix Ex Taq™ II (Takara, Osaka, Japan). PCR was performed under the following conditions: denaturation at 95 °C (10 min), followed by 40 cycles at 95 °C (10 s) and 60 °C (60 s). The cDNA was amplified at 95 °C (10 s), 60 °C (60 s), and 95 °C (15 s). Following this, the temperature was gradually increased from 60 °C to 99 °C to establish the melting curve of the PCR products. The circRNA expression levels were calculated using the 2^−ΔΔCt^ method. The level of β-actin expression was used to normalize the data. The outward-facing primers for each selected circRNA were designed for targeting the backsplicing junction sites and are shown in Table 1.

**TABLE 1:** Sense and anti-sense primers for RT-qPCR.

Genes name	Primer	Sequences (5’-3’)
chr2:72945232-72960247- (hsa_circ_0009043)	Forward Reverse	TGGAGAGCATCCGCAAACAT GTCGACACTGCTTCAGCTCT
chr3:114069121-114070725- (hsa_circ_0005332)	Forward Reverse	CACACAGGTGACATCAGTTGC CGAGCACGGAATTGCTGAAG
chr10:126370176-126370948- (hsa_circ_0000267)	Forward Reverse	ACGACAAGAAGGTCGGTGTT GCCATCTGTCATTTTCTGTGCC
chr5:142416761-142437312+ (hsa_circ_0074368)	Forward Reverse	TGTTCATCGGCTCCCAGAGA CCCTTGCTCGTTGATCCCTC
chr5:76342172-76344097+ (hsa_circ_0008620)	Forward Reverse	TTGGCCCCCATGTATTAGAGT TCAAATCCTTCCAATTGTAGCAG
β-actin	Forward Reverse	CATGTACGTTGCTATCCAGGC CTCCTTAATGTCACGCACGAT

### 2.8 Statistical processing

All results were presented as mean ± SD. All statistical analyses were performed using GraphPad Prism 8.0 (GraphPad Software, CA, United States). Student’s t-test was used to compare the statistical difference between different groups.

The bioinformatics statistical analysis of RNA-seq data was carried out as follows: the edgeR software package was used to standardize the gene expression counts, specifically employing the TMM (weighted trimmed mean) method to correct for compositional differences between samples. Based on this, a statistical model was constructed using the negative binomial generalized linear model to evaluate differences in the expression of each gene among different groups. To control the false-positive rate, the p-values obtained from all hypothesis tests were corrected using the Benjamini–Hochberg method. *p < 0.05 indicated statistically significant difference.

## 3 Results

### 3.1 Influence of PRP activation on the concentration of secreted EVs

**FIGURE 1:**
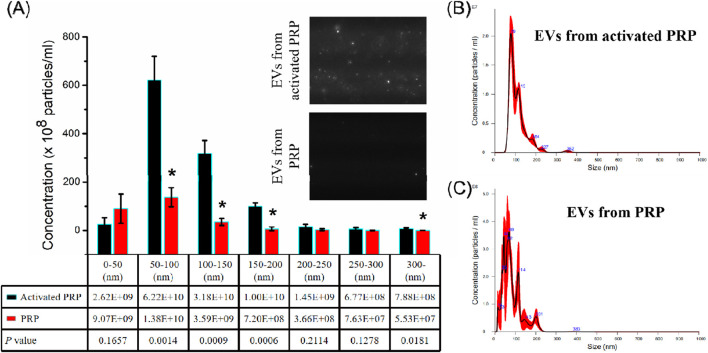
Concentration and size distribution of EVs from activated PRP and PRP using NTA. **(A)** Differences in concentrations between EVs from activated PRP and EVs from PRP across different diameters. **(B)** Particle size distributions of EVs from activated PRP. **(C)** Particle size distributions of EVs from PRP. *p < 0.05.

The PRP-EV populations from PRP and activated PRP were analyzed by NTA, and their differences were compared. As shown in Figure 1, for both PRP and activated PRP, the majority of the secreted particles were measured to be within 300 nm in diameter, which is the standard diameter for EVs. In all the tables of the article, the “Gene name” column includes both the chromosome location and the circBase number. In addition, the total concentration of EVs from PRP was nearly 2.77 ± 0.80 × 10^10^ particles/mL, and that from activated PRP was nearly 1.10 ± 0.11 × 10^11^ particles/mL. This result indicated that the number of secreted EVs significantly increased after activation by thrombin—a five- to tenfold increase in EV number compared to that from PRP—particularly those with diameters ranging from 50 to 200 nm. Altogether, the NTA results demonstrated that thrombin could activate PRP, and it notably promoted the formation and secretion of EVs, particularly those with diameters ranging from 50 to 200 nm.

### 3.2 Characterization of PRP-EVs and plasma-EVs

**FIGURE 2:**
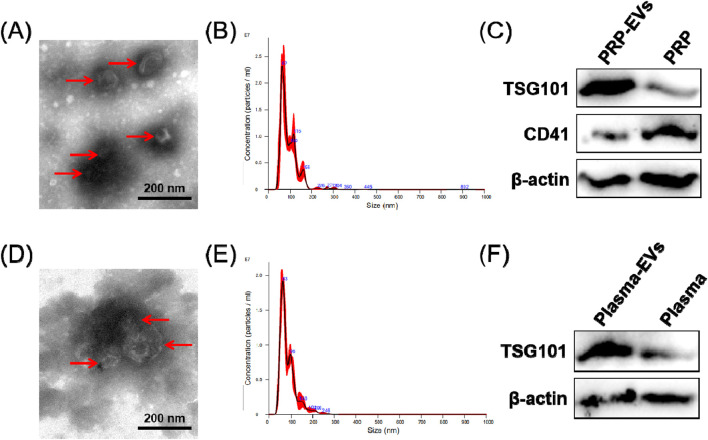
Characterization of EVs derived from PRP (PRP-EVs) and EVs derived from plasma (plasma-EVs). **(A)** Micromorphology of PRP-EVs using transmission electron microscopy (TEM). **(B)** Particle size distributions of PRP-EVs based on NTA. **(C)** Western blot analysis of the EV surface marker TSG101 and the platelet marker CD41 in PRP-EVs. **(D)** Micromorphology of plasma-EVs using TEM. **(E)** Particle size distributions of plasma-EVs based on NTA. **(F)** Western blot analysis of the EV surface marker TSG101 in plasma-EVs. β-Actin was only used as the reference for the load quantity.

In order to characterize the PRP-EVs and plasma-EVs, TEM, Western blotting, and NTA were performed. Under TEM observation, PRP-EVs and plasma-EVs exhibited an irregular cup- or sphere-shaped micromorphology (approximately 100 nm in diameter), indicating the common morphology of EVs, as shown in Figures 2A,D. The NTA results indicated that both PRP-EVs and plasma-EVs had a narrow size distribution, with diameters ranging from 20 to 200 nm, as shown in Figures 2B,E. As shown in Figure 2C, the results of Western blotting demonstrated that PRP-EVs showed enrichment of the EV marker TSG101 and low expression of the platelet marker CD41. Similarly, plasma-EVs also showed a high expression of the EV marker TSG101, as shown in Figure 2F. However, both PRP-EVs and plasma-EVs exhibited a low level of the internal reference protein β-actin, as shown in Figures 2C,F. Altogether, the above results confirmed these nanoparticles isolated through ultracentrifugation to be EVs.

### 3.3 Differential expression profiles of circRNAs in PRP-EVs

**FIGURE 3:**
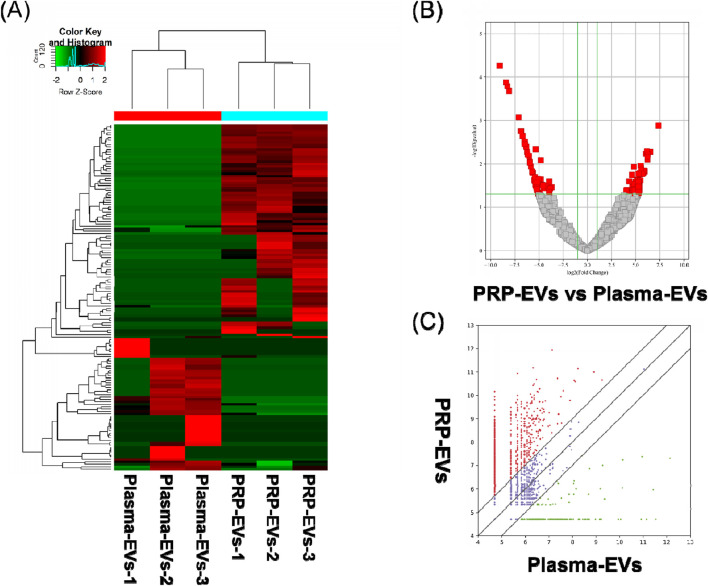
Differentially expressed levels of circRNAs in PRP-EVs compared with those of plasma-EVs. **(A)** Hierarchical cluster analysis of differentially expressed circRNAs (fold change ≥ 2; p ≤ 0.05). Green represents a low expression level, and red represents a high expression level. **(B)** Volcano plot of differentially expressed circRNAs. The red dots represent the differentially expressed circRNAs with statistical significance (fold change ≥ 2.0; p ≤ 0.05). **(C)** Scatter plot of differentially expressed circRNAs. The upper and lower oblique lines represent the fold change of ±2.0, respectively. The red dots represent the upregulated circRNAs, and the green dots represent the downregulated circRNAs in PRP-EVs.

The RNA-seq raw reads of each sample are presented in Supplementary Table S1. In this study, a total of 144 significantly differentially expressed circRNAs in PRP-EVs in comparison with plasma-EVs were identified using the criteria of fold change ≥ 2.0 and p ≤ 0.05. Among these circRNAs, 89 were upregulated (Supplementary Table S2), and 55 were downregulated (Supplementary Table S3). In addition, the top 10 upregulated circRNAs and top 10 downregulated circRNAs according to the fold change are listed in Table 2. Hierarchical cluster analysis illustrated all the differentially expressed circRNA profiles in PRP-EVs compared with plasma-EVs, as shown in Figure 3A. The volcano plot and scatter plot were used to identify and visualize the differentially expressed circRNAs in PRP-EVs, as shown in Figures 3B,C. Furthermore, the upregulated host genes of differentially expressed circRNAs were mainly located in the exonic regions (88.76%), rather than intronic regions (4.49%), sense overlapping regions (5.62%), and antisense regions (1.12%) (Supplementary Table S2). In contrast, the downregulated host genes were mainly located in the intronic regions (49.09%), rather than exonic regions (16.36%), sense overlapping regions (27.27%), antisense regions (3.64%), and intergenic regions (3.64%).

**TABLE 2:** Top 10 up-regulated and top 10 down-regulated circRNAs in PRP-EVs.

Genomic location	Log FC	P-value	CircBase ID	Gene name	Catalog	Regulation
chrX:139865340-139866824+	7.372714	0.001317	hsa_circ_0001946	CDR1	antisense	up
chr7:6840579-6841154-	6.499321	0.005304	hsa_circ_0007135	CCZ1B	exonic	up
chr21:16386665-16415895-	6.263895	0.006136	hsa_circ_0004771	NRIP1	exonic	up
chr16:30495148-30495584+	6.235946	0.007853	hsa_circ_0000690	ITGAL	exonic	up
chr3:145838899-145842016-	6.230007	0.006881	hsa_circ_0122319	PLOD2	exonic	up
chr6:31239376-31324219+	6.210923	0.005779	novel	RPL3P2	sense overlapping	up
chr19:6702138-6702590-	6.164770	0.005173	hsa_circ_0002130	C3	exonic	up
chr3:149563798-149639014+	6.068016	0.005867	hsa_circ_0001346	RNF13	exonic	up
chr18:45391430-45423180-	5.928080	0.014908	hsa_circ_0000847	SMAD2	exonic	up
chr7:5963018-5963593+	5.831571	0.014523	hsa_circ_0007177	CCZ1	exonic	up
chrM:14131-15754-	-9.052668	0.000055	novel	JA760602	sense overlapping	down
chrM:14131-15754+	-8.417218	0.000132	novel	MTND5	sense overlapping	down
chrM:14068-14923+	-8.262566	0.000163	novel	MTND5	sense overlapping	down
chrM:14056-14263-	-8.081547	0.000212	novel	JA760602	intronic	down
chrM:4198-6296-	-7.098869	0.000847	novel	G087360	intronic	down
chrM:7749-8685-	-6.850636	0.001756	novel	G087360	intronic	down
chrM:14068-14446-	-6.842494	0.001780	novel	JA760602	intronic	down
chrM:4198-6296+	-6.700063	0.002291	novel	G087361	intronic	down
chr10:116879949-116889297+	-6.463991	0.003077	hsa_circ_0020093	ATRNL1	exonic	down
chrM:14414-14923-	-6.450791	0.003561	novel	JA760602	intronic	down

### 3.4 GO and KEGG pathway analyses of differentially expressed circRNAs in PRP-EVs

**FIGURE 4:**
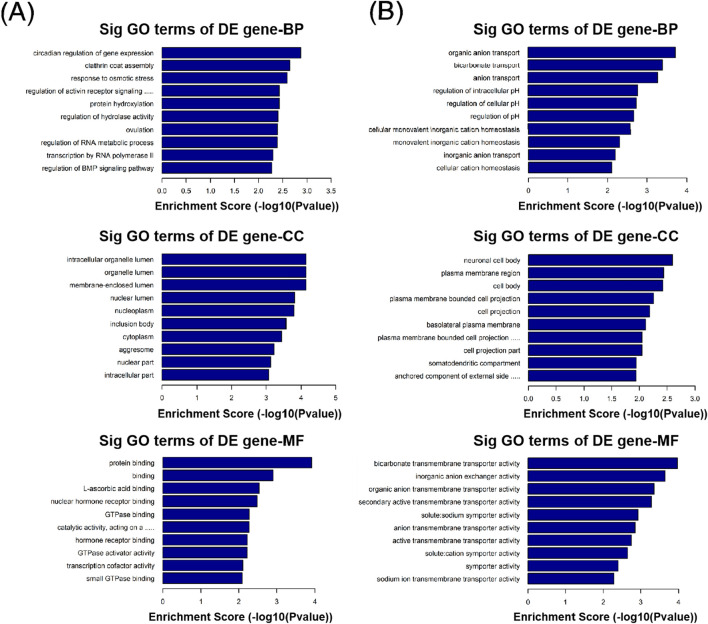
Gene Ontology (GO) analysis of differentially expressed circRNAs with top 10 enrichment scores, including biological process (BP), cell component (CC), and molecular function (MF). **(A)** GO analysis of upregulated circRNAs. **(B)** GO analysis of downregulated circRNAs.

GO function analysis was performed on circRNA-derived host genes with significant differences to speculate the potential functions of these differentially expressed circRNAs. GO analysis includes three different aspects: biological process (BP), cell component (CC), and molecular function (MF). In this study, 105 GO BP terms, 34 GO CC terms, and 49 GO MF terms for upregulated circRNAs and 81 GO BP terms, 36 GO CC terms, and 25 GO MF terms for downregulated circRNAs were found to be enriched (Supplementary Tables S4–S9). The top 10 enriched GO terms are presented in Figure 4. With regard to BP, the upregulated circRNAs were mainly associated with regulation of protein functions and cellular signal transduction, such as clathrin coat assembly, protein hydroxylation, regulation of hydrolase activity, regulation of the activin receptor signaling pathway, and regulation of the BMP signaling pathway. In contrast, the downregulated circRNAs were mainly related to ion transport and homeostasis, such as organic anion transport, bicarbonate transport, anion transport, cellular monovalent inorganic cation homeostasis, monovalent inorganic cation homeostasis, and cellular cation homeostasis. With regard to CC, the top three enriched GO terms for the upregulated circRNAs were membrane-enclosed lumen, organelle lumen, and intracellular organelle lumen. The top three enriched GO terms for the downregulated circRNAs were neuronal cell body, plasma membrane region, and cell body. With regard to MF, the top three enriched GO terms for the upregulated circRNAs were protein binding, binding, and L-ascorbic acid binding. The top three enriched GO terms for the downregulated circRNAs were bicarbonate transmembrane transporter activity, inorganic anion exchanger activity, and organic anion transmembrane transporter activity.

**FIGURE 5:**
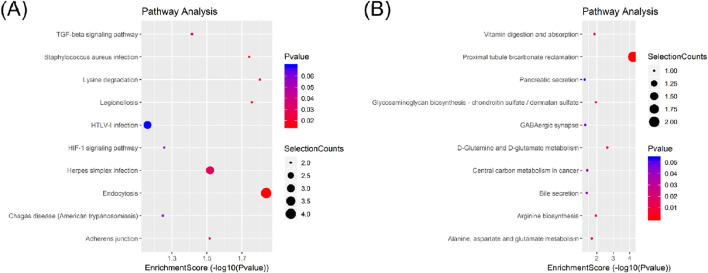
Dot plot of Kyoto Encyclopedia of Genes and Genomes (KEGG) pathway analysis of differentially expressed circRNAs with top 10 enrichment scores. **(A)** KEGG pathway analysis of upregulated circRNAs. **(B)** KEGG pathway analysis of downregulated circRNAs.

**FIGURE 6:**
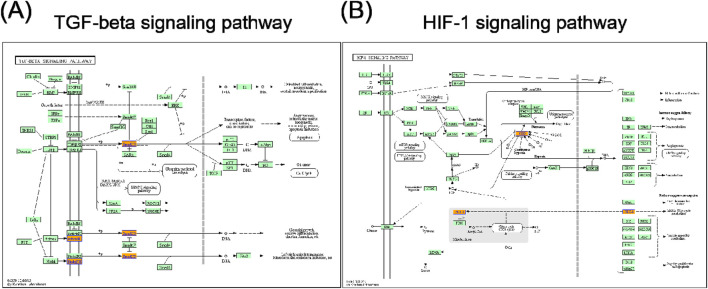
**(A)** TGF-β signaling pathway and **(B)** HIF-1 signaling pathway. Orange nodes are associated with upregulated genes, and green nodes represent no significance.

In the KEGG pathway analysis, a total of 15 pathways for upregulated circRNAs and 11 pathways for downregulated circRNAs were predicted (Supplementary Tables S10, S11). Based on the enrichment score [−log_10_ (p-value)], the dot plot of top 10 pathways of differentially expressed circRNAs in PRP-EVs are presented in Figure 5. Among these pathways, we predicted that the upregulated circRNAs in PRP-EVs were associated with the TGF-β signaling pathway and HIF-1 signaling pathway, which are key growth factor-related signaling pathways involved in promoting tissue repair and regeneration, as shown in Figures 6A,B.

### 3.5 Validation of differentially expressed circRNAs in PRP-EVs by RT-qPCR

**FIGURE 7:**
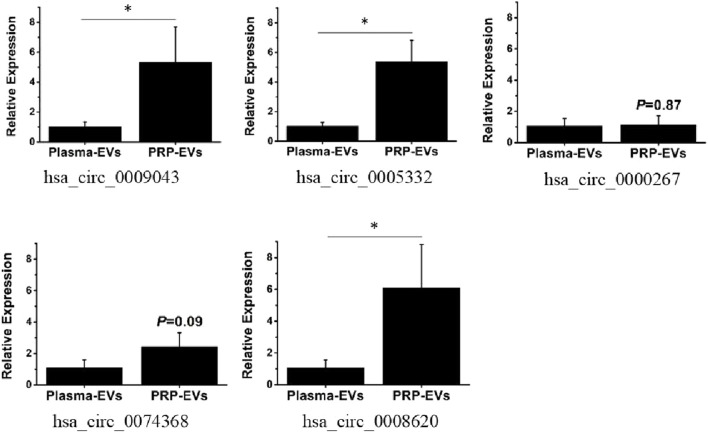
RT-qPCR validation of five randomly selected differentially expressed circRNAs. The fold change was defined using the 2^−ΔΔCt^ method. (*p < 0.05 indicates statistically significant difference).

To verify the accuracy of the RNA-seq data, five differentially expressed circRNAs with the greatest fold changes and of same origin were randomly selected for RT-qPCR verification. The relative quantitative results are presented in Figure 7. The expression levels of all selected circRNAs in PRP-EVs were higher than those in plasma-EVs. Three candidate circRNAs of the greatest fold change, namely, chr2:72945232-72960247− (hsa_circ_0009043), chr3:114069121-114070725− (hsa_circ_0005332), and chr5:76342172-76344097+ (hsa_circ_0008620), were significantly upregulated in PRP-EVs compared with plasma-EVs (P < 0.05), which were in good agreement with previous RNA-seq results. Although no significant differences were found (P > 0.05), the other candidate circRNAs chr10:126370176-126370948- (hsa_circ_0000267) and chr5:142416761-142437312+ (hsa_circ_0074368) also exhibited concordant results with previous RNA-seq results. Altogether, the results of RT-qPCR demonstrated that the circRNA expression profiles obtained from RNA-seq were reliable.

## 4 Discussion

Unlike many linear RNAs, circRNAs are a class of single-stranded closed RNA molecules formed by specific backsplicing events within precursor mRNA (pre-mRNA), which is among the most important members in the long non-coding RNA (lncRNA) family (Liang et al., 2024; Kim, 2024). circRNAs are widely expressed in all types of eukaryotic cells. Hansen et al. (2013) first reported the role of circRNAs as miRNA- or RNA-binding protein sponges. Other studies also revealed that circRNAs serve as regulators of splicing and transcriptional events, modifiers of parental gene expression, and templates for protein translation (Legnini et al., 2017; Zhang and Wang, 2024). Furthermore, given the conserved and stable features across different species, most circRNAs holds promise as potential diagnostic and predictive biomarkers with clinical implications (Verwilt and Vromman, 2024).

Over the past two decades, the discovery of the role of EVs in cell-to-cell communication is considered a revolutionary concept in cell biology. EVs are fundamental for cell-to-cell communication through regulation of a diverse range of biological functions (Janowska-Wi et al., 2001; Bai et al., 2019). EVs act as bioactive cargos that transfer various growth factors, proteins, lipids, nucleic acids, miRNAs, and lncRNAs (Wang et al., 2019). In 2015, Li et al. (2015) first reported the abundant presence of circRNAs in exosomes (Verwilt and Vromman, 2024). Advances in high-throughput sequencing and bioinformatics techniques have opened new avenues for understanding the biological functions of circRNAs in EVs. As described in Section 1, PRP-EVs have great potential for promoting tissue repair and regeneration (Tao et al., 2017b; Guo et al., 2017; Zhang et al., 2019). More importantly, based on the biological functions of circRNAs, we speculate that circRNAs in PRP-EVs may play an important role in mediating their biological effects. However, the expression profiles and potential biological functions of circRNAs in PRP-EVs remain unknown.

Therefore, in this study, we first measured the total EV populations from PRP and activated PRP using NTA and demonstrated that PRP activation by thrombin could significantly enhance the formation and secretion of EVs, particularly those with diameters ranging from 50 to 200 nm. A previous study has demonstrated that activation is an important step in effective PRP protocols because it releases various growth factors that enhance cell mitosis, angiogenesis, chondrogenesis, and chemotaxis (Wu et al., 2021). In addition, many agonists can activate PRP, including CaCl_2_, autologous thrombin, CaCl_2_ with thrombin, and collagen type I (Cavallo et al., 2016). Furthermore, Aatonen et al. (2014) also found that PRP activation by thrombin and collagen, lipopolysaccharide, or Ca^2+^ ionophore could differentially regulate the quantity and quality of secreted EVs. Our study further demonstrated thrombin as an effective activator to promote the formation and secretion of PRP-EVs, which contributed to the clinical application of PRP-EVs.

Then we used RNA-seq techniques and bioinformatics analysis to explore the roles and potential functions of altered circRNAs in PRP-EVs. A total of 144 circRNAs were significantly differentially expressed in PRP-EVs, including 89 upregulated and 55 downregulated circRNAs compared with those in plasma-EVs. To the best of our knowledge, this is the first report to assess the expression levels of circRNAs in PRP-EVs, which may provide novel insights into the understanding of the biological functions of PRP-EVs. In addition, five differentially expressed circRNAs with relatively high fold changes were randomly selected for validation by RT-qPCR, and the results were consistent with the expression profiles from RNA-seq. These five candidate circRNAs were derived from the exonic host genes, namely, *EXOC6B*, *ZBTB20*, *FAM53B*, *ARHGAP26*, and *AGGF1*, respectively. Based on the gene information of NCBI (https://www.ncbi.nlm.nih.gov/), their encoded proteins may play an important role in cell growth, polarity, and migration; neurogenesis, glucose homeostasis, and postnatal growth; cell proliferation; GTPase activation; and proliferation of endothelial cells, respectively.

In addition, GO and KEGG pathway analyses were also performed to preliminarily predict the roles and potential mechanisms of altered circRNAs in PRP-EVs. GO analysis of biological process indicated that the enriched circRNAs were mainly associated with regulation of protein functions and cellular signal transduction, such as clathrin coat assembly, protein hydroxylation, regulation of hydrolase activity, regulation of the activin receptor signaling pathway, and regulation of the BMP signaling pathway. Furthermore, GO analysis of molecular function revealed that these enriched circRNAs were mainly associated with the binding of protein, nuclear hormone receptor binding, GTPase binding, GTPase activator activity, small GTPase binding, and GTPase regulator activity, which might be involved in the physiological regulation of important intracellular proteins. Thus, we suspected that these altered circRNAs might be relevant to the physiological processes of cell genesis and development, including the assembly, modification, and metabolism of key proteins and the transduction of important cellular signals. To some extent, it might help us explain the potential mechanisms of biological functions of PRP-EVs in promoting tissue repair and regeneration. KEGG pathway analysis showed the enriched biological pathways that the differentially expressed circRNAs participated in. Of these pathways, the TGF-β signaling pathway and the HIF-1 signaling pathway were considered the key signaling pathways underlying the biological functions of PRP-EVs in promoting tissue repair and regeneration.

TGF-β is a type of multifunctional growth factor that controls a variety of fundamental aspects of cellular behaviors. The TGF-β signaling pathway, an important growth factor-related signaling pathway, has pleiotropic functions in regulating cell proliferation, migration, differentiation, apoptosis, extracellular matrix production, angiogenesis, and immune response (Horbelt et al., 2012; Neuzillet et al., 2015). It plays a critical role in early embryonic development and in regulating tissue homeostasis, direct wound healing processes, and reproduction in adults (Nolan and Thompson, 2014; Wakefield and Hill, 2013). The TGF-β pathway includes SMAD pathways, evoking a transcriptional response, and non-SMAD pathways, which result in the activation of MAP kinases, JNK, Akt/PKB, small GTPases, and other factors (Horbelt et al., 2012). Moreover, TGF-β signaling can also affect other important biological signaling pathways, including Erk, Wnt, SAPK/JNK, PI3K/AKT, and p38 MAPK pathways (Xie et al., 2018). Thus, due to the functions in cell proliferation and survival, TGF-β signaling pathway and other related signaling pathways may contribute to the potential functions of PRP-EVs in promoting tissue repair and regeneration.

Tissue repair and regeneration are highly complex overlapping events, the most important of which is the adaptive response of hypoxic stress and angiogenesis. HIF-1 induces the synthesis of proteins that promote metabolic changes in the cells of hypoxic tissues and induce angiogenesis to re-establish an adequate oxygen supply (Mitroshina and Vedunova, 2024). In addition, HIF-1 is central to the regulation of apoptosis and cell survival, cell adhesion and extracellular matrix turnover, cytoskeletal structure, cell motility, epithelial homeostasis, erythropoiesis and iron metabolism, metabolic homeostasis, and pH regulation (Fujii et al., 2024). It is evident that the HIF-1 signaling pathway is a key pathway regulating various cellular behaviors, particularly vascular remodeling. Therefore, we believed that the HIF-1 signaling pathway may be an important regulator of protective and regenerative mechanisms following hypoxic stress after tissue injury.

miRNAs are well-known to be highly conserved and have important functions in negatively regulating target mRNAs (Billi et al., 2024; Nalbant and Akkaya-Ulum, 2024). In addition, accumulating evidence indicates that circRNAs bind to the downstream miRNAs and consequently repress their functions (Zhang and Wang, 2024; Maatouk et al., 2024). To explore the role of differentially expressed circRNAs in PRP-EVs, we constructed the circRNA–miRNA–mRNA interaction network to predict the functions and pathways of circRNAs. Five candidate circRNAs validated by RT-qPCR were selected, and the top five predicted targeted miRNAs for each differentially expressed circRNA and the top eight predicted targeted mRNAs for each miRNA were identified. The circRNA–miRNA–mRNA interaction network provides an important reference for screening the key regulatory sites and pathways to reveal the functional mechanisms of PRP-EVs in our future research.

Several previous studies have found that circRNAs can perform various physiological functions. In the study on colorectal cancer, Hsa-circ-PVT1 was discovered to bind miR-145 as a “molecular sponge,” thereby upregulating the expression of the *MAPK1* gene and promoting the proliferation, invasion, and angiogenesis of endometrial stromal cells (Yang et al., 2018). Another study on colorectal cancer showed that the exosome circCOL1A1 derived from cancer cells could also promote angiogenesis by recruiting the EIF4A3 protein and activating the Smad2/3 signaling pathway (Hu et al., 2023). Additionally, a study on the treatment of ischemic diseases found that hsa_circ_0093884 in exosomes derived from endothelial progenitor cells could promote therapeutic neovascularization through the miR-145/SIRT1 pathway. These cases illustrate that circRNAs can be transmitted through exosomes and act as messengers to regulate angiogenesis (Zhao et al., 2024). circRNAs have also been reported to play a role in wound healing in the skin. Studies have shown that circRNAs are involved in regulating the proliferation and migration of cells (such as vascular endothelial cells and keratinocytes) related to wound healing. In the study of keloids (a disease related to abnormal wound healing), researchers identified differentially expressed circRNAs during wound healing and constructed their competitive endogenous RNA (ceRNA) network, suggesting that circRNAs may affect the healing quality by regulating the expression of related genes (Yu et al., 2022).

However, there are still some limitations in our study. First, the potential functions of these circRNAs differentially expressed in PRP-EVs were predicted using bioinformatics analysis and without further validation in any recipient cells. In our future studies, we will focus on these potential roles and functions for further elucidation. Second, due to the ethical limitations of human samples, only three samples in each group were enrolled in this study. Following this preliminary verification, an increased sample size will improve the scientific rigor of future studies.

## 5 Conclusion

To the best of our knowledge, this is the first study to provide evidence that circRNAs are differentially expressed in PRP-EVs. GO, KEGG pathway, and circRNA–miRNA–mRNA interaction network analyses were also performed to predict the roles and potential functions of altered circRNAs in PRP-EVs. For the first time, we explained the potential mechanisms underlying the biological functions of PRP-EVs in terms of the altered circRNAs. We believe that this study may lay the groundwork for the clinical application of PRP-EVs and provide possible novel targets for further research.

## Data Availability

The raw data supporting the conclusions of this article will be made available by the authors, without undue reservation.
