# Supplementary material for: Altered circRNAs: a novel potential mechanism for the functions of extracellular vesicles derived from platelet-rich plasma
Source: Front Bioinform. 2026 Jan 8;5:1690932. doi: 10.3389/fbinf.2025.1690932 (PMC12823818; doi:10.3389/fbinf.2025.1690932)
Supplement: Supplementary file 1 [file Table1.docx]

Supplementary Table 1. Reads statistics

| **Sample** | **Raw Reads** | **Clean Reads** | **Mapped Reads** | **circRNA Number** |
| --- | --- | --- | --- | --- |
| PRP-EVs 1 | 103367626 | 102997102 | 89749264 | 2612 |
| PRP-EVs 2 | 80254068 | 80071194 | 72267420 | 3807 |
| PRP-EVs 3 | 95908420 | 95781818 | 87512128 | 3633 |
| Plasma-EVs 1 | 83129710 | 83058026 | 70500896 | 1892 |
| Plasma-EVs 2 | 88811678 | 88771316 | 80587024 | 3021 |
| Plasma-EVs 3 | 83597758 | 83514968 | 74611190 | 2049 |
