# Supplementary material for: Altered circRNAs: a novel potential mechanism for the functions of extracellular vesicles derived from platelet-rich plasma
Source: Front Bioinform. 2026 Jan 8;5:1690932. doi: 10.3389/fbinf.2025.1690932 (PMC12823818; doi:10.3389/fbinf.2025.1690932)
Supplement: Supplementary file 3 [file Table2.docx]

Supplementary Table 2. All the up-regulated circRNAs in PRP-EVs

| **CircRNA ID** | **Log FC** | **P-value** | **CircBase ID** | **Gene name** | **Catalog** |
| --- | --- | --- | --- | --- | --- |
| chrX:139865340-139866824+ | 7.372714 | 0.001317 | hsa_circ_0001946 | CDR1 | antisense |
| chr7:6840579-6841154- | 6.499321 | 0.005304 | hsa_circ_0007135 | CCZ1B | exonic |
| chr21:16386665-16415895- | 6.263895 | 0.006136 | hsa_circ_0004771 | NRIP1 | exonic |
| chr16:30495148-30495584+ | 6.235946 | 0.007853 | hsa_circ_0000690 | ITGAL | exonic |
| chr3:145838899-145842016- | 6.230007 | 0.006881 | hsa_circ_0122319 | PLOD2 | exonic |
| chr6:31239376-31324219+ | 6.210923 | 0.005779 | novel | RPL3P2 | sense overlapping |
| chr19:6702138-6702590- | 6.164770 | 0.005173 | hsa_circ_0002130 | C3 | exonic |
| chr3:149563798-149639014+ | 6.068016 | 0.005867 | hsa_circ_0001346 | RNF13 | exonic |
| chr18:45391430-45423180- | 5.928080 | 0.014908 | hsa_circ_0000847 | SMAD2 | exonic |
| chr7:5963018-5963593+ | 5.831571 | 0.014523 | hsa_circ_0007177 | CCZ1 | exonic |
| chr2:148653870-148657467+ | 5.797137 | 0.016339 | hsa_circ_0001073 | ACVR2A | exonic |
| chr16:30490412-30490782+ | 5.701719 | 0.015890 | novel | ITGAL | exonic |
| chr8:142264088-142264728- | 5.684941 | 0.016936 | hsa_circ_0001829 | SLC45A4 | exonic |
| chr18:8718422-8720494+ | 5.566593 | 0.013817 | hsa_circ_0000825 | MTCL1 | exonic |
| chr19:13039156-13039661- | 5.556439 | 0.012611 | hsa_circ_0000896 | FARSA | exonic |
| chr2:72945232-72960247- | 5.540428 | 0.017022 | hsa_circ_0009043 | EXOC6B | exonic |
| chr3:114069121-114070725- | 5.524855 | 0.010972 | hsa_circ_0005332 | ZBTB20 | exonic |
| chr14:34394824-34400421- | 5.481650 | 0.023983 | hsa_circ_0101692 | EGLN3 | sense overlapping |
| chr1:35824526-35827390+ | 5.461335 | 0.017229 | hsa_circ_0011536 | ZMYM4 | exonic |
| chr6:16326625-16328701- | 5.439642 | 0.016346 | hsa_circ_0007132 | ATXN1 | exonic |
| chr7:74171152-74172333+ | 5.385986088 | 0.029670062 | hsa_circ_0006672 | GTF2I | exonic |
| chr1:231928640-231954263+ | 5.37994651 | 0.040197561 | novel | DISC1 | intronic |
| chr2:89082251-89092011+ | 5.363669414 | 0.030980422 | novel | ANKRD36BP2 | exonic |
| chr1:29481208-29481422- | 5.349992449 | 0.04229128 | hsa_circ_0006602 | SRSF4 | exonic |
| chr17:57808782-57816308+ | 5.336580321 | 0.034940833 | hsa_circ_0006508 | VMP1 | exonic |
| chr7:30590252-30601744- | 5.331513 | 0.027671 | hsa_circ_0001693 | LOC401320 | exonic |

| chr3:119219542-119232566+ | 5.321520 | 0.044322 | hsa_circ_0006884 | TIMMDC1 | exonic |
| --- | --- | --- | --- | --- | --- |
| chr19:30476130-30477324+ | 5.314314 | 0.026976 | hsa_circ_0000921 | URI1 | exonic |
| chr3:138289160-138290198- | 5.308787 | 0.033194 | hsa_circ_0002468 | CEP70 | exonic |
| chr10:5836848-5842668- | 5.293434 | 0.023784 | hsa_circ_0002665 | GDI2 | exonic |
| chr7:129760589-129762042+ | 5.292680 | 0.034000 | hsa_circ_0002190 | KLHDC10 | exonic |
| chr2:168920010-168931741- | 5.289212 | 0.016815 | hsa_circ_0003279 | STK39 | exonic |
| chr1:151139410-151139890+ | 5.284821 | 0.024918 | hsa_circ_0000128 | SCNM1 | exonic |
| chr2:63206323-63223901+ | 5.279788 | 0.025936 | hsa_circ_0005552 | EHBP1 | exonic |
| chr3:142455221-142467302+ | 5.279315 | 0.047360 | hsa_circ_0001345 | TRPC1 | exonic |
| chr1:1158624-1159348- | 5.262222 | 0.031566 | hsa_circ_0000002 | SDF4 | exonic |
| chr2:10559860-10560261+ | 5.238257 | 0.039740 | hsa_circ_0000976 | HPCAL1 | exonic |
| chr3:195686054-195686957- | 5.230928 | 0.034056 | hsa_circ_0002174 | GSE61474_XLOC_045249 | intronic |
| chr9:37424842-37426651+ | 5.230736 | 0.022992 | hsa_circ_0001861 | GRHPR | exonic |
| chr8:128902835-128903244+ | 5.229638 | 0.028981 | hsa_circ_0001821 | PVT1 | exonic |
| chr3:56626998-56628056+ | 5.214019 | 0.032359 | hsa_circ_0001313 | CCDC66 | exonic |
| chr19:45528587-45528995+ | 5.208461 | 0.025864 | hsa_circ_0008590 | RELB | exonic |
| chr17:45695716-45696530+ | 5.188817 | 0.048552 | hsa_circ_0004622 | NPEPPS | exonic |
| chr10:126370176-126370948- | 5.187350 | 0.025878 | hsa_circ_0000267 | FAM53B | exonic |
| chr4:40596275-40598817- | 5.164463 | 0.040970 | hsa_circ_0126347 | RBM47 | intronic |
| chr14:55168780-55169298+ | 5.148695 | 0.046215 | hsa_circ_0004846 | SAMD4A | exonic |
| chr7:155457869-155473602+ | 5.130144 | 0.033796 | hsa_circ_0001771 | RBM33 | exonic |
| chr2:201721405-201721708- | 5.129030 | 0.025103 | hsa_circ_0004001 | CLK1 | exonic |
| chr18:8076453-8088849+ | 5.128524 | 0.027757 | hsa_circ_0006114 | PTPRM | exonic |
| chr2:173435454-173460751+ | 5.092466 | 0.039782 | hsa_circ_0006006 | PDK1 | exonic |
| chr6:108984658-108986092+ | 5.088448 | 0.030154 | hsa_circ_0006404 | FOXO3 | exonic |
| chr12:2929252-2930958+ | 5.057254 | 0.034980 | hsa_circ_0000374 | ITFG2 | exonic |
| chr7:104678573-104681470+ | 5.044837 | 0.036484 | hsa_circ_0007395 | KMT2E | Exonic |

| chr4:36230204-36231267- | 4.664946 | 0.048253 | hsa_circ_0069399 | ARAP2 | exonic |
| --- | --- | --- | --- | --- | --- |
| chr19:1032391-1032695+ | 4.655363 | 0.042219 | hsa_circ_0004891 | CNN2 | exonic |
| chr15:32814649-32825569- | 4.634329 | 0.035035 | novel | WHAMMP1 | sense overlapping |
| chr2:168920010-168986268- | 4.624224 | 0.018152 | hsa_circ_0005882 | STK39 | exonic |
| chr11:120347370-120348235+ | 4.616414 | 0.049761 | hsa_circ_0002089 | ARHGEF12 | exonic |
| chr7:24663285-24690331+ | 4.550081 | 0.048881 | hsa_circ_0001685 | MPP6 | exonic |
| chr15:101906401-101910728- | 4.493048 | 0.048498 | hsa_circ_0037096 | PCSK6 | exonic |
| chr5:124036707-124036962- | 4.333661 | 0.025400 | hsa_circ_0001523 | ZNF608 | exonic |
| chr4:37633007-37640126- | 4.055047 | 0.039712 | hsa_circ_0001400 | RELL1 | exonic |
| chr7:99621042-99621930+ | 4.031541 | 0.041918 | hsa_circ_0001727 | ZKSCAN1 | exonic |
