# Supplementary material for: Altered circRNAs: a novel potential mechanism for the functions of extracellular vesicles derived from platelet-rich plasma
Source: Front Bioinform. 2026 Jan 8;5:1690932. doi: 10.3389/fbinf.2025.1690932 (PMC12823818; doi:10.3389/fbinf.2025.1690932)
Supplement: Supplementary file 4 [file Table5.docx]

Supplementary Table 10: The KEGG pathways analysis for the up-regulated circRNAs

| **Pathway ID** | **Definition** | **Fisher P-value** | **Enrichment Score** | **Gene Ratio** |
| --- | --- | --- | --- | --- |
| hsa04144 | Endocytosis | 0.014476 | 1.839357 | 0.153846 |
| hsa00310 | Lysine degradation | 0.015740 | 1.802987 | 0.076923 |
| hsa05134 | Legionellosis | 0.017508 | 1.756762 | 0.076923 |
| hsa05150 | Staphylococcus aureus infection | 0.018115 | 1.741954 | 0.076923 |
| hsa05168 | Herpes simplex infection | 0.030329 | 1.518146 | 0.115385 |
| hsa04520 | Adherens junction | 0.030503 | 1.515659 | 0.076923 |
| hsa04350 | TGF-beta signaling pathway | 0.038489 | 1.414667 | 0.076923 |
| hsa04066 | HIF-1 signaling pathway | 0.055568 | 1.255172 | 0.076923 |
| hsa05142 | Chagas disease (American trypanosomiasis) | 0.056531 | 1.247715 | 0.076923 |
| hsa05166 | HTLV-I infection | 0.069186 | 1.159984 | 0.115385 |
| hsa04110 | Cell cycle | 0.076959 | 1.113739 | 0.076923 |
| hsa04068 | FoxO signaling pathway | 0.087939 | 1.055817 | 0.076923 |
| hsa04514 | Cell adhesion molecules (CAMs) | 0.097047 | 1.013018 | 0.076923 |
| hsa04550 | Signaling pathways regulating pluripotency of stem cells | 0.097047 | 1.013018 | 0.076923 |
| hsa00630 | Glyoxylate and dicarboxylate metabolism | 0.099266 | 1.003199 | 0.038462 |
