# Supplementary material for: Altered circRNAs: a novel potential mechanism for the functions of extracellular vesicles derived from platelet-rich plasma
Source: Front Bioinform. 2026 Jan 8;5:1690932. doi: 10.3389/fbinf.2025.1690932 (PMC12823818; doi:10.3389/fbinf.2025.1690932)
Supplement: Supplementary file 5 [file Table3.docx]

| **CircRNA ID** | **Log FC** | **P-value** | **CircBase ID** | **Gene name** | **Catalog** |
| --- | --- | --- | --- | --- | --- |
| chrM:14131-15754- | -9.052668 | 0.000055 | novel | JA760602 | sense overlapping |
| chrM:14131-15754+ | -8.417218 | 0.000132 | novel | MTND5 | sense overlapping |
| chrM:14068-14923+ | -8.262566 | 0.000163 | novel | MTND5 | sense overlapping |
| chrM:14056-14263- | -8.081547 | 0.000212 | novel | JA760602 | intronic |
| chrM:4198-6296- | -7.098869 | 0.000847 | novel | G087360 | intronic |
| chrM:7749-8685- | -6.850636 | 0.001756 | novel | G087360 | intronic |
| chrM:14068-14446- | -6.842494 | 0.001780 | novel | JA760602 | intronic |
| chrM:4198-6296+ | -6.700063 | 0.002291 | novel | G087361 | intronic |
| chr10:116879949-116889297+ | -6.463991 | 0.003077 | hsa_circ_0020093 | ATRNL1 | exonic |
| chrM:14414-14923- | -6.450791 | 0.003561 | novel | JA760602 | intronic |
| chrM:14213-15778- | -6.303768 | 0.004027 | novel | JA760602 | sense overlapping |
| chrM:13861-15754- | -6.243591 | 0.005369 | novel | JA760602 | sense overlapping |
| chrM:14068-15382+ | -6.176361 | 0.006103 | novel | MTND5 | sense overlapping |
| chrM:5483-6492- | -6.035127 | 0.006621 | novel | G087360 | intronic |
| chrM:14074-14394- | -5.960229 | 0.009094 | novel | JA760602 | intronic |
| chrM:13856-14217- | -5.942430 | 0.009414 | novel | JA760602 | intronic |
| chrM:14068-14827+ | -5.839648 | 0.011460 | novel | MTND5 | sense overlapping |
| chr7:2472529-2472892+ | -5.692956 | 0.015061 | novel | CHST12 | sense overlapping |
| chrM:13856-14421- | -5.657706 | 0.016178 | novel | JA760602 | intronic |
| chr1:45925067-45925864- | -5.474956 | 0.023411 | novel | TESK2 | intronic |
| chrM:14056-16359- | -5.465719 | 0.023832 | novel | JA760602 | sense overlapping |
| chrM:7749-8685+ | -5.450192 | 0.024555 | novel | OK/SW-cl.16 | sense overlapping |
| chr4:49313820-49319959- | -5.420573 | 0.020096 | novel | G061014 | intronic |
| chr17:76840825-76841186+ | -5.352298 | 0.029599 | novel | G035951 | sense overlapping |
| chrM:14068-15403- | -5.348955 | 0.029783 | novel | JA760602 | intronic |
| chrM:14056-14377+ | -5.346108 | 0.004674 | novel | MTND5 | sense overlapping |
| chrM:14068-15382- | -5.315097 | 0.031700 | novel | JA760602 | intronic |
| chr3:63141694-63146475+ | -5.315097 | 0.031700 | novel | - | intergenic |

Supplementary Table 3. All the down-regulated circRNAs in PRP-EVs

| chr15:57730183-57754090+ | -5.256701 | 0.034932 | hsa_circ_0035432 | CGNL1 | exonic |
| --- | --- | --- | --- | --- | --- |
| chrM:5426-5929+ | -5.218321 | 0.037901 | novel | G087361 | intronic |
| chr3:177365664-177366349- | -5.218321 | 0.037901 | novel | LINC00578 | antisense |
| chr5:9890487-9891713- | -5.218321 | 0.037901 | novel | LOC285692 | intronic |
| chrM:14414-14923+ | -5.209969 | 0.022322 | novel | cytochrome b | sense overlapping |
| chrM:5483-6492+ | -5.199897 | 0.039175 | novel | G087361 | intronic |
| chrM:13847-14413- | -5.193185 | 0.026904 | novel | JA760602 | intronic |
| chr5:101612950-101613137- | -5.181235 | 0.040503 | novel | SLCO4C1 | intronic |
| chr12:51858305-51867619- | -5.181235 | 0.040503 | novel | SLC4A8 | antisense |
| chrM:14131-15286- | -5.181235 | 0.040503 | novel | JA760602 | intronic |
| chr2:191765290-191769893+ | -5.080982 | 0.047846 | hsa_circ_0006662 | GLS | exonic |
| chr5:99662584-99662955+ | -5.070739 | 0.049352 | novel | G066858 | intronic |
| chrM:2227-8822- | -5.070739 | 0.049352 | novel | G087360 | intronic |
| chr4:72385598-72386636+ | -5.070739 | 0.049352 | novel | SLC4A4 | intronic |
| chrM:14056-15335- | -5.042711 | 0.022488 | novel | JA760602 | intronic |
| chr5:57793761-57794541+ | -4.902742 | 0.045763 | novel | - | intergenic |
| chrM:14056-14377- | -4.813253 | 0.008255 | novel | JA760602 | intronic |
| chr6:51875107-51900519- | -4.795129 | 0.022681 | novel | PKHD1 | exonic |
| chr22:46096162-46136418+ | -4.672839 | 0.030681 | hsa_circ_0001246 | ATXN10 | exonic |
| chr14:31404369-31425448- | -4.547169 | 0.031435 | hsa_circ_0031446 | STRN3 | exonic |
| chrX:79544405-79565732- | -4.542784 | 0.036653 | hsa_circ_0140637 | CHMP1B2P | exonic |
| chrM:14068-14446+ | -4.233221 | 0.033356 | novel | MTND5 | sense overlapping |
| chr6:51695659-51735437- | -3.946004 | 0.043983 | novel | PKHD1 | exonic |
| chrM:14068-14413- | -3.937757 | 0.025383 | novel | JA760602 | intronic |
| chr10:17024483-17061982- | -3.918042 | 0.048436 | novel | CUBN | exonic |
| chrM:14068-14413+ | -3.843636 | 0.036981 | novel | MTND5 | sense overlapping |
| chrM:14068-14923- | -3.691633 | 0.034854 | novel | JA760602 | intronic |
