# Supplementary material for: Altered circRNAs: a novel potential mechanism for the functions of extracellular vesicles derived from platelet-rich plasma
Source: Front Bioinform. 2026 Jan 8;5:1690932. doi: 10.3389/fbinf.2025.1690932 (PMC12823818; doi:10.3389/fbinf.2025.1690932)
Supplement: Supplementary file 6 [file Table4.docx]

Supplementary Table 4. The biological process (BP) terms of GO analysis for the up-regulated circRNAs

| **GO ID** | **Terms** | **P-value** | **Enrichment Score** | **Gene Ratio** |
| --- | --- | --- | --- | --- |
| GO:0032922 | circadian regulation of gene expression | 0.001351 | 2.869189 | 0.042857 |
| GO:0048268 | clathrin coat assembly | 0.002276 | 2.642806 | 0.028571 |
| GO:0006970 | response to osmotic stress | 0.002594 | 2.585971 | 0.042857 |
| GO:0018126 | protein hydroxylation | 0.003716 | 2.429932 | 0.028571 |
| GO:0032925 | regulation of activin receptor signaling pathway | 0.003716 | 2.429932 | 0.028571 |
| GO:0051336 | regulation of hydrolase activity | 0.003930 | 2.405584 | 0.171429 |
| GO:0030728 | ovulation | 0.004043 | 2.393254 | 0.028571 |
| GO:0051252 | regulation of RNA metabolic process | 0.004051 | 2.392402 | 0.371429 |
| GO:0006366 | transcription by RNA polymerase II | 0.005006 | 2.300505 | 0.257143 |
| GO:0030510 | regulation of BMP signaling pathway | 0.005315 | 2.274493 | 0.042857 |
| GO:0006357 | regulation of transcription by RNA polymerase II | 0.005338 | 2.272617 | 0.242857 |
| GO:0045648 | positive regulation of erythrocyte differentiation | 0.006276 | 2.202329 | 0.028571 |
| GO:0071470 | cellular response to osmotic stress | 0.007119 | 2.147552 | 0.028571 |
| GO:0016070 | RNA metabolic process | 0.007359 | 2.133160 | 0.414286 |
| GO:0090100 | positive regulation of transmembrane receptor protein serine/threonine kinase signaling pathway | 0.007748 | 2.110834 | 0.042857 |
| GO:0071456 | cellular response to hypoxia | 0.008050 | 2.094211 | 0.057143 |
| GO:0030218 | erythrocyte differentiation | 0.008831 | 2.053972 | 0.042857 |
| GO:0000578 | embryonic axis specification | 0.008951 | 2.048121 | 0.028571 |
| GO:0036294 | cellular response to decreased oxygen levels | 0.009065 | 2.042652 | 0.057143 |
| GO:0051345 | positive regulation of hydrolase activity | 0.009745 | 2.011233 | 0.114286 |
| GO:0034101 | erythrocyte homeostasis | 0.010742 | 1.968912 | 0.042857 |
| GO:0051056 | regulation of small GTPase mediated signal transduction | 0.011088 | 1.955158 | 0.071429 |
| GO:0007368 | determination of left/right symmetry | 0.011516 | 1.938717 | 0.042857 |
| GO:0071453 | cellular response to oxygen levels | 0.011689 | 1.932216 | 0.057143 |
| GO:0018107 | peptidyl-threonine phosphorylation | 0.011780 | 1.928844 | 0.042857 |
| GO:0001666 | response to hypoxia | 0.012031 | 1.919694 | 0.071429 |
| GO:0032924 | activin receptor signaling pathway | 0.012048 | 1.919090 | 0.028571 |
| GO:0043484 | regulation of RNA splicing | 0.012049 | 1.919065 | 0.042857 |
| GO:0043087 | regulation of GTPase activity | 0.012075 | 1.918102 | 0.085714 |

| GO:0090092 | regulation of transmembrane receptor protein serine/threonine kinase signaling pathway | 0.012230 | 1.912566 | 0.057143 |
| --- | --- | --- | --- | --- |
| GO:0019219 | regulation of nucleobase-containing compound metabolic process | 0.012522 | 1.902329 | 0.371429 |
| GO:0022602 | ovulation cycle process | 0.012603 | 1.899509 | 0.0285719 |
| GO:0036293 | response to decreased oxygen levels | 0.013175 | 1.880258 | 0.071429 |
| GO:0016192 | vesicle-mediated transport | 0.013235 | 1.878266 | 0.214286 |
| GO:0000122 | negative regulation of transcription by RNA polymerase II | 0.013582 | 1.867023 | 0.114286 |
| GO:0032648 | regulation of interferon-beta production | 0.013748 | 1.861769 | 0.028571 |
| GO:0045646 | regulation of erythrocyte differentiation | 0.013748 | 1.861769 | 0.028571 |
| GO:0009855 | determination of bilateral symmetry | 0.014025 | 1.853097 | 0.042857 |
| GO:0009799 | specification of symmetry | 0.014321 | 1.844012 | 0.042857 |
| GO:0048872 | homeostasis of number of cells | 0.014760 | 1.830918 | 0.057143 |
| GO:0032608 | interferon-beta production | 0.014935 | 1.825784 | 0.028571 |
| GO:0035023 | regulation of Rho protein signal transduction | 0.015857 | 1.799768 | 0.042857 |
| GO:0090304 | nucleic acid metabolic process | 0.016467 | 1.783388 | 0.428571 |
| GO:0070482 | response to oxygen levels | 0.017043 | 1.768450 | 0.071429 |
| GO:0002262 | myeloid cell homeostasis | 0.017819 | 1.749126 | 0.042857 |
| GO:0018210 | peptidyl-threonine modification | 0.017819 | 1.749126 | 0.042857 |
| GO:0009628 | response to abiotic stimulus | 0.019228 | 1.716073 | 0.142857 |
| GO:0006887 | exocytosis | 0.021282 | 1.671986 | 0.114286 |
| GO:0051865 | protein autoubiquitination | 0.022210 | 1.653447 | 0.028571 |
| GO:0043547 | positive regulation of GTPase activity | 0.022646 | 1.645007 | 0.071429 |
| GO:0090287 | regulation of cellular response to growth factor stimulus | 0.022867 | 1.640791 | 0.057143 |
| GO:0048511 | rhythmic process | 0.023968 | 1.620364 | 0.057143 |
| GO:0002285 | lymphocyte activation involved in immune response | 0.026540 | 1.576101 | 0.042857 |
| GO:0030509 | BMP signaling pathway | 0.026540 | 1.576101 | 0.042857 |
| GO:0009880 | embryonic pattern specification | 0.026680 | 1.573818 | 0.028571 |
| GO:0019915 | lipid storage | 0.026680 | 1.573818 | 0.028571 |
| GO:0042698 | ovulation cycle | 0.026680 | 1.573818 | 0.028571 |
| GO:0010468 | regulation of gene expression | 0.030080 | 1.521721 | 0.385714 |
| GO:0071772 | response to BMP | 0.031849 | 1.496909 | 0.042857 |
| GO:0071773 | cellular response to BMP stimulus | 0.031849 | 1.496909 | 0.042857 |
| GO:0051253 | negative regulation of RNA metabolic process | 0.032542 | 1.487552 | 0.142857 |
| GO:0050790 | regulation of catalytic activity | 0.033070 | 1.480562 | 0.214286 |
| GO:0023051 | regulation of signaling | 0.034021 | 1.468247 | 0.3 |
| GO:0045944 | positive regulation of transcription by RNA polymerase II | 0.034206 | 1.465901 | 0.128571 |

| GO:0002274 | myeloid leukocyte activation | 0.037323 | 1.428027 | 0.085714 |
| --- | --- | --- | --- | --- |
| GO:2000177 | regulation of neural precursor cell proliferation | 0.037474 | 1.426270 | 0.028571 |
| GO:0034764 | positive regulation of transmembrane transport | 0.038181 | 1.418148 | 0.042857 |
| GO:0002863 | positive regulation of inflammatory response to antigenic stimulus | 0.038963 | 1.409348 | 0.014286 |
| GO:0006828 | manganese ion transport | 0.038963 | 1.409348 | 0.014286 |
| GO:0007440 | foregut morphogenesis | 0.038963 | 1.409348 | 0.014286 |
| GO:0031053 | primary miRNA processing | 0.038963 | 1.409348 | 0.014286 |
| GO:0048340 | paraxial mesoderm morphogenesis | 0.038963 | 1.409348 | 0.014286 |
| GO:0051956 | negative regulation of amino acid transport | 0.038963 | 1.409348 | 0.014286 |
| GO:0097499 | protein localization to non-motile cilium | 0.038963 | 1.409348 | 0.014286 |
| GO:1901033 | positive regulation of response to reactive oxygen species | 0.038963 | 1.409348 | 0.014286 |
| GO:0006006 | glucose metabolic process | 0.039209 | 1.406616 | 0.042857 |
| GO:0000288 | nuclear-transcribed mRNA catabolic process, deadenylation-dependent decay | 0.040167 | 1.396134 | 0.028571 |
| GO:0006725 | cellular aromatic compound metabolic process | 0.041137 | 1.385772 | 0.457143 |
| GO:0009966 | regulation of signal transduction | 0.042007 | 1.376682 | 0.271429 |
| GO:0045892 | negative regulation of transcription, DNA-templated | 0.042051 | 1.376219 | 0.128572 |
| GO:0007623 | circadian rhythm | 0.042375 | 1.372889 | 0.042858 |
| GO:0090527 | actin filament reorganization | 0.042776 | 1.368801 | 0.014286 |
| GO:1903960 | negative regulation of anion transmembrane transport | 0.042776 | 1.368801 | 0.014286 |
| GO:2000574 | regulation of microtubule motor activity | 0.042776 | 1.368801 | 0.014286 |
| GO:0006139 | nucleobase-containing compound metabolic process | 0.043652 | 1.359996 | 0.442857 |
| GO:0007178 | transmembrane receptor protein serine/threonine kinase signaling pathway | 0.044383 | 1.352781 | 0.057143 |
| GO:0006351 | transcription, DNA-templated | 0.045528 | 1.341723 | 0.314286 |
| GO:0007266 | Rho protein signal transduction | 0.046227 | 1.335100 | 0.042857 |
| GO:0002864 | regulation of acute inflammatory response to antigenic stimulus | 0.046574 | 1.331856 | 0.014286 |
| GO:0016554 | cytidine to uridine editing | 0.046574 | 1.331856 | 0.014286 |
| GO:0019471 | 4-hydroxyproline metabolic process | 0.046574 | 1.331856 | 0.014286 |
| GO:0034982 | mitochondrial protein processing | 0.046574 | 1.331856 | 0.014286 |
| GO:0045475 | locomotor rhythm | 0.046574 | 1.331856 | 0.014286 |
| GO:2000650 | negative regulation of sodium ion transmembrane transporter activity | 0.046574 | 1.331856 | 0.014286 |

| GO:0010467 | gene expression | 0.046708 | 1.330607 | 0.428571 |
| --- | --- | --- | --- | --- |
| GO:0045639 | positive regulation of myeloid cell differentiation | 0.046723 | 1.330469 | 0.028571 |
| GO:0097659 | nucleic acid-templated transcription | 0.047505 | 1.323265 | 0.314286 |
| GO:0042119 | neutrophil activation | 0.047508 | 1.323232 | 0.071429 |
| GO:0030111 | regulation of Wnt signaling pathway | 0.047666 | 1.321793 | 0.057143 |
| GO:0046903 | secretion | 0.047803 | 1.320547 | 0.157143 |
| GO:0009952 | anterior/posterior pattern specification | 0.047930 | 1.319392 | 0.042857 |
| GO:0002446 | neutrophil mediated immunity | 0.048185 | 1.317085 | 0.071429 |
| GO:0032774 | RNA biosynthetic process | 0.049423 | 1.306070 | 0.314286 |
| GO:0036230 | granulocyte activation | 0.049557 | 1.304895 | 0.071429 |
| GO:0009798 | axis specification | 0.049645 | 1.304121 | 0.028571 |

Supplementary Table 5. The cell component (CC) terms of GO analysis for the up-regulated circRNAs

| **GO ID** | **Terms** | **P-value** | **Enrichment Score** | **Gene Ratio** |
| --- | --- | --- | --- | --- |
| GO:0031974 | membrane-enclosed lumen | 0.000073 | 4.137420 | 0.493151 |
| GO:0043233 | organelle lumen | 0.000073 | 4.137420 | 0.493151 |
| GO:0070013 | intracellular organelle lumen | 0.000073 | 4.137420 | 0.493151 |
| GO:0031981 | nuclear lumen | 0.000154 | 3.812436 | 0.410959 |
| GO:0005654 | nucleoplasm | 0.000162 | 3.791046 | 0.369864 |
| GO:0016234 | inclusion body | 0.000270 | 3.568246 | 0.054795 |
| GO:0005737 | cytoplasm | 0.000358 | 3.446574 | 0.808220 |
| GO:0016235 | aggresome | 0.000588 | 3.230958 | 0.041096 |
| GO:0044428 | nuclear part | 0.000734 | 3.134195 | 0.410959 |
| GO:0044424 | intracellular part | 0.000843 | 3.074275 | 0.917809 |
| GO:0005829 | cytosol | 0.001285 | 2.890985 | 0.438357 |
| GO:0043229 | intracellular organelle | 0.001487 | 2.827632 | 0.835616 |
| GO:0043227 | membrane-bounded organelle | 0.001542 | 2.811928 | 0.835616 |
| GO:0043226 | organelle | 0.001711 | 2.766773 | 0.876712 |
| GO:0005622 | intracellular | 0.002105 | 2.676737 | 0.917808 |
| GO:0005634 | nucleus | 0.004114 | 2.385776 | 0.547945 |
| GO:0044444 | cytoplasmic part | 0.004863 | 2.313089 | 0.671233 |
| GO:0044437 | vacuolar part | 0.006828 | 2.165718 | 0.095890 |
| GO:0043231 | intracellular membrane-bounded organelle | 0.007274 | 2.138198 | 0.726027 |
| GO:0016604 | nuclear body | 0.008199 | 2.086254 | 0.109589 |
| GO:0044446 | intracellular organelle part | 0.009006 | 2.045481 | 0.630137 |
| GO:0044451 | nucleoplasm part | 0.010952 | 1.960487 | 0.136986 |
| GO:0044422 | organelle part | 0.015981 | 1.796390 | 0.630137 |
| GO:0016607 | nuclear speck | 0.016597 | 1.779966 | 0.068493 |
| GO:0005768 | endosome | 0.020827 | 1.681375 | 0.109589 |
| GO:0005774 | vacuolar membrane | 0.022844 | 1.641228 | 0.068493 |
| GO:0005905 | clathrin-coated pit | 0.028233 | 1.549236 | 0.027397 |
| GO:0005773 | vacuole | 0.030947 | 1.509378 | 0.095890 |
| GO:0072546 | ER membrane protein complex | 0.038372 | 1.415987 | 0.013699 |
| GO:0042405 | nuclear inclusion body | 0.045870 | 1.338470 | 0.013699 |
| GO:0000323 | lytic vacuole | 0.047475 | 1.323534 | 0.082192 |
| GO:0005764 | lysosome | 0.047475 | 1.323534 | 0.082192 |
| GO:0035578 | azurophil granule lumen | 0.049270 | 1.307416 | 0.027397 |
| GO:0000176 | nuclear exosome (RNase complex) | 0.049598 | 1.304540 | 0.013699 |

Supplementary Table 6. The molecular function (MF) terms of GO analysis for the up-regulated circRNAs

| **GO ID** | **Terms** | **P-value** | **Enrichment Score** | **Gene Ratio** |
| --- | --- | --- | --- | --- |
| GO:0005515 | protein binding | 0.000122 | 3.912413 | 0.859155 |
| GO:0005488 | binding | 0.001241 | 2.906227 | 0.971831 |
| GO:0031418 | L-ascorbic acid binding | 0.002926 | 2.533792 | 0.028169 |
| GO:0035257 | nuclear hormone receptor binding | 0.003274 | 2.484864 | 0.056338 |
| GO:0051020 | GTPase binding | 0.005299 | 2.275804 | 0.112676 |
| GO:0140096 | catalytic activity, acting on a protein | 0.005357 | 2.271042 | 0.253521 |
| GO:0051427 | hormone receptor binding | 0.005980 | 2.223295 | 0.056338 |
| GO:0005096 | GTPase activator activity | 0.006021 | 2.220324 | 0.070423 |
| GO:0003712 | transcription cofactor activity | 0.007829 | 2.106293 | 0.098592 |
| GO:0031267 | small GTPase binding | 0.008123 | 2.090286 | 0.098592 |
| GO:0030695 | GTPase regulator activity | 0.009067 | 2.042533 | 0.070423 |
| GO:0019899 | enzyme binding | 0.009093 | 2.041274 | 0.239437 |
| GO:0045296 | cadherin binding | 0.010032 | 1.998626 | 0.070423 |
| GO:0050681 | androgen receptor binding | 0.013096 | 1.882859 | 0.028169 |
| GO:0050839 | cell adhesion molecule binding | 0.014137 | 1.849650 | 0.084507 |
| GO:0016706 | oxidoreductase activity, acting on paired donors, with incorporation or reduction of molecular oxygen, 2-oxoglutarate as one donor, and incorporation of one atom each of oxygen into both donors | 0.014284 | 1.845164 | 0.028169 |
| GO:0003714 | transcription corepressor activity | 0.014504 | 1.838519 | 0.056338 |
| GO:0000989 | transcription factor activity, transcription factor binding | 0.015043 | 1.822669 | 0.098592 |
| GO:0060589 | nucleoside-triphosphatase regulator activity | 0.015086 | 1.821412 | 0.070423 |
| GO:0000988 | transcription factor activity, protein binding | 0.015635 | 1.805890 | 0.098592 |
| GO:0043167 | ion binding | 0.017653 | 1.753174 | 0.478873 |
| GO:0008047 | enzyme activator activity | 0.018585 | 1.730840 | 0.084507 |
| GO:0001047 | core promoter binding | 0.019872 | 1.701750 | 0.042254 |
| GO:0043621 | protein self-association | 0.020171 | 1.695280 | 0.028169 |
| GO:0003682 | chromatin binding | 0.021918 | 1.659194 | 0.084507 |
| GO:0030374 | ligand-dependent nuclear receptor transcription coactivator activity | 0.031813 | 1.497397 | 0.028169 |
| GO:0048029 | monosaccharide binding | 0.031813 | 1.497397 | 0.028169 |
| GO:0017048 | Rho GTPase binding | 0.033035 | 1.481029 | 0.042254 |
| GO:0046332 | SMAD binding | 0.036159 | 1.441785 | 0.028169 |
| GO:0008134 | transcription factor binding | 0.039264 | 1.406004 | 0.084507 |

Supplementary Table 7. The biological process (BP) terms of GO analysis for the down-regulated circRNAs

| **GO ID** | **Terms** | **P-value** | **Enrichment Score** | **Gene Ratio** |
| --- | --- | --- | --- | --- |
| GO:0015711 | organic anion transport | 0.000191 | 3.719520 | 0.333333 |
| GO:0015701 | bicarbonate transport | 0.000394 | 3.404037 | 0.166667 |
| GO:0006820 | anion transport | 0.000526 | 3.279041 | 0.333333 |
| GO:0051453 | regulation of intracellular pH | 0.001677 | 2.775383 | 0.166667 |
| GO:0030641 | regulation of cellular pH | 0.001826 | 2.738471 | 0.166667 |
| GO:0006885 | regulation of pH | 0.002101 | 2.677549 | 0.166667 |
| GO:0030004 | cellular monovalent inorganic cation homeostasis | 0.002615 | 2.582455 | 0.166667 |
| GO:0055067 | monovalent inorganic cation homeostasis | 0.004953 | 2.305095 | 0.166667 |
| GO:0015698 | inorganic anion transport | 0.006381 | 2.195132 | 0.166667 |
| GO:0030003 | cellular cation homeostasis | 0.007854 | 2.104884 | 0.25 |
| GO:0030208 | dermatan sulfate biosynthetic process | 0.008129 | 2.089944 | 0.083333 |
| GO:0034384 | high-density lipoprotein particle clearance | 0.008129 | 2.089944 | 0.083333 |
| GO:0006873 | cellular ion homeostasis | 0.008344 | 2.078602 | 0.25 |
| GO:0001967 | suckling behavior | 0.008804 | 2.055317 | 0.083333 |
| GO:0002087 | regulation of respiratory gaseous exchange by neurological system process | 0.008804 | 2.055317 | 0.083333 |
| GO:0030205 | dermatan sulfate metabolic process | 0.008804 | 2.055317 | 0.083333 |
| GO:0033147 | negative regulation of intracellular estrogen receptor signaling pathway | 0.009478 | 2.023268 | 0.083333 |
| GO:0043650 | dicarboxylic acid biosynthetic process | 0.009478 | 2.023268 | 0.083333 |
| GO:0006814 | sodium ion transport | 0.009646 | 2.015646 | 0.166667 |
| GO:0050651 | dermatan sulfate proteoglycan biosynthetic process | 0.010152 | 1.993440 | 0.083333 |
| GO:0055080 | cation homeostasis | 0.010733 | 1.969272 | 0.25 |
| GO:0044065 | regulation of respiratory system process | 0.010826 | 1.965546 | 0.083333 |
| GO:0050655 | dermatan sulfate proteoglycan metabolic process | 0.010826 | 1.965546 | 0.083333 |
| GO:0098771 | inorganic ion homeostasis | 0.011280 | 1.947687 | 0.25 |
| GO:0009084 | glutamine family amino acid biosynthetic process | 0.012171 | 1.914664 | 0.083333 |
| GO:0042953 | lipoprotein transport | 0.012843 | 1.891318 | 0.083333 |
| GO:0044872 | lipoprotein localization | 0.012843 | 1.891318 | 0.083333 |
| GO:0098656 | anion transmembrane transport | 0.013225 | 1.878600 | 0.166667 |
| GO:0055082 | cellular chemical homeostasis | 0.013679 | 1.863946 | 0.25 |
| GO:0050801 | ion homeostasis | 0.013968 | 1.854870 | 0.25 |
| GO:0009235 | cobalamin metabolic process | 0.014857 | 1.828055 | 0.083333 |
| GO:0006541 | glutamine metabolic process | 0.015528 | 1.808885 | 0.083333 |
| GO:0042359 | vitamin D metabolic process | 0.015528 | 1.808885 | 0.083333 |
| GO:0043576 | regulation of respiratory gaseous exchange | 0.015528 | 1.808885 | 0.083333 |
| GO:0030206 | chondroitin sulfate biosynthetic process | 0.016868 | 1.772943 | 0.083333 |

| GO:0006811 | ion transport | 0.018782 | 1.726259 | 0.333333 |
| --- | --- | --- | --- | --- |
| GO:0051260 | protein homooligomerization | 0.019176 | 1.717252 | 0.166667 |
| GO:0070207 | protein homotrimerization | 0.019542 | 1.709025 | 0.083333 |
| GO:0003016 | respiratory system process | 0.020210 | 1.694437 | 0.083333 |
| GO:0009065 | glutamine family amino acid catabolic process | 0.020210 | 1.694437 | 0.083333 |
| GO:0050650 | chondroitin sulfate proteoglycan biosynthetic process | 0.020210 | 1.694437 | 0.083333 |
| GO:0019725 | cellular homeostasis | 0.021545 | 1.666652 | 0.25 |
| GO:0006536 | glutamate metabolic process | 0.022210 | 1.653450 | 0.083333 |
| GO:0033144 | negative regulation of intracellular steroid hormone receptor signaling pathway | 0.022210 | 1.653450 | 0.083333 |
| GO:0042592 | homeostatic process | 0.023465 | 1.629577 | 0.333333 |
| GO:0051180 | vitamin transport | 0.023542 | 1.628166 | 0.083333 |
| GO:0060271 | cilium assembly | 0.024538 | 1.610165 | 0.166667 |
| GO:0071702 | organic substance transport | 0.024579 | 1.609437 | 0.416667 |
| GO:0043252 | sodium-independent organic anion transport | 0.024871 | 1.6043027 | 0.083333 |
| GO:0044782 | cilium organization | 0.026191 | 1.581854 | 0.166667 |
| GO:0010824 | regulation of centrosome duplication | 0.026199 | 1.581709 | 0.083333 |
| GO:0033146 | regulation of intracellular estrogen receptor signaling pathway | 0.026199 | 1.581709 | 0.083333 |
| GO:0030204 | chondroitin sulfate metabolic process | 0.027526 | 1.560260 | 0.083333 |
| GO:0014047 | glutamate secretion | 0.028188 | 1.549930 | 0.083333 |
| GO:0050654 | chondroitin sulfate proteoglycan metabolic process | 0.028851 | 1.539845 | 0.083333 |
| GO:0006775 | fat-soluble vitamin metabolic process | 0.029512 | 1.529996 | 0.083333 |
| GO:0051181 | cofactor transport | 0.029512 | 1.529996 | 0.083333 |
| GO:0046394 | carboxylic acid biosynthetic process | 0.031695 | 1.499005 | 0.166667 |
| GO:0016053 | organic acid biosynthetic process | 0.031835 | 1.497096 | 0.166667 |
| GO:0070206 | protein trimerization | 0.034133 | 1.466823 | 0.083333 |
| GO:0048878 | chemical homeostasis | 0.035900 | 1.444909 | 0.25 |
| GO:0051058 | negative regulation of small GTPase mediated signal transduction | 0.036107 | 1.442404 | 0.083333 |
| GO:0046605 | regulation of centrosome cycle | 0.036765 | 1.434570 | 0.083333 |
| GO:0034381 | plasma lipoprotein particle clearance | 0.038078 | 1.419328 | 0.083333 |
| GO:0051898 | negative regulation of protein kinase B signaling | 0.038078 | 1.419328 | 0.083333 |
| GO:0030520 | intracellular estrogen receptor signaling pathway | 0.038734 | 1.411909 | 0.083333 |
| GO:0033013 | tetrapyrrole metabolic process | 0.038734 | 1.411909 | 0.083333 |
| GO:0015800 | acidic amino acid transport | 0.040045 | 1.397456 | 0.083333 |
| GO:0030166 | proteoglycan biosynthetic process | 0.040045 | 1.397456 | 0.083333 |
| GO:1901607 | alpha-amino acid biosynthetic process | 0.042008 | 1.376671 | 0.083333 |

Supplementary Table 8. The cell component (CC) terms of GO analysis for the down-regulated circRNAs

| **GO ID** | **Terms** | **P-value** | **Enrichment Score** | **Gene Ratio** |
| --- | --- | --- | --- | --- |
| GO:0043025 | neuronal cell body | 0.002575 | 2.589293 | 0.25 |
| GO:0098590 | plasma membrane region | 0.003632 | 2.439882 | 0.333333 |
| GO:0044297 | cell body | 0.003755 | 2.425439 | 0.25 |
| GO:0120025 | plasma membrane bounded cell projection part | 0.005538 | 2.256609 | 0.416667 |
| GO:0042995 | cell projection | 0.006505 | 2.186746 | 0.416667 |
| GO:0016323 | basolateral plasma membrane | 0.007694 | 2.113858 | 0.166667 |
| GO:0044463 | cell projection part | 0.008975 | 2.046986 | 0.333333 |
| GO:0120038 | plasma membrane bounded cell projection part | 0.008975 | 2.046986 | 0.333333 |
| GO:0036477 | somatodendritic compartment | 0.011419 | 1.942386 | 0.25 |
| GO:0031362 | anchored component of external side of plasma membrane | 0.011494 | 1.939512 | 0.083333 |
| GO:0000159 | protein phosphatase type 2A complex | 0.013398 | 1.872949 | 0.083333 |
| GO:0009897 | external side of plasma membrane | 0.014000 | 1.853886 | 0.166667 |
| GO:0031233 | intrinsic component of external side of plasma membrane | 0.014666 | 1.833695 | 0.083333 |
| GO:0032809 | neuronal cell body membrane | 0.014666 | 1.833695 | 0.083333 |
| GO:0044298 | cell body membrane | 0.014666 | 1.833695 | 0.083333 |
| GO:0016324 | apical plasma membrane | 0.015228 | 1.817351 | 0.166667 |
| GO:0031226 | intrinsic component of plasma membrane | 0.020148 | 1.695770 | 0.333333 |
| GO:0005765 | lysosomal membrane | 0.020921 | 1.679415 | 0.166667 |
| GO:0098852 | lytic vacuole membrane | 0.020921 | 1.679415 | 0.166667 |
| GO:0045177 | apical part of cell | 0.022153 | 1.654573 | 0.166667 |
| GO:0044459 | plasma membrane part | 0.024761 | 1.606227 | 0.416667 |
| GO:0005774 | vacuolar membrane | 0.027873 | 1.554813 | 0.166667 |
| GO:0030173 | integral component of Golgi membrane | 0.027884 | 1.554648 | 0.083333 |
| GO:0046658 | anchored component of plasma membrane | 0.030383 | 1.517369 | 0.083333 |
| GO:0031228 | intrinsic component of Golgi membrane | 0.031007 | 1.508542 | 0.083333 |
| GO:0008287 | protein serine/threonine phosphatase complex | 0.031630 | 1.499895 | 0.083333 |
| GO:1903293 | phosphatase complex | 0.031630 | 1.499895 | 0.083333 |
| GO:0031526 | brush border membrane | 0.032876 | 1.483117 | 0.083333 |
| GO:0005886 | plasma membrane | 0.034828 | 1.458066 | 0.583333 |
| GO:0035577 | azurophil granule membrane | 0.036605 | 1.436456 | 0.083333 |
| GO:0071944 | cell periphery | 0.038719 | 1.412075 | 0.583333 |
| GO:0043005 | neuron projection | 0.039435 | 1.404120 | 0.25 |
| GO:0098552 | side of membrane | 0.040752 | 1.389853 | 0.166667 |
| GO:0005905 | clathrin-coated pit | 0.042174 | 1.374955 | 0.083333 |
| GO:0016459 | myosin complex | 0.044640 | 1.350280 | 0.083333 |
| GO:0030425 | dendrite | 0.049862 | 1.302233 | 0.166667 |

Supplementary Table 9. The molecular function (MF) terms of GO analysis for the down-regulated circRNAs

| **GO ID** | **Terms** | **P-value** | **Enrichment Score** | **Gene Ratio** |
| --- | --- | --- | --- | --- |
| GO:0015106 | bicarbonate transmembrane transporter activity | 0.000108 | 3.968202 | 0.166667 |
| GO:0005452 | inorganic anion exchanger activity | 0.000224 | 3.650339 | 0.166667 |
| GO:0008514 | organic anion transmembrane transporter activity | 0.000442 | 3.354242 | 0.25 |
| GO:0015291 | secondary active transmembrane transporter activity | 0.000520 | 3.284176 | 0.25 |
| GO:0015370 | solute:sodium symporter activity | 0.001188 | 2.925223 | 0.166667 |
| GO:0008509 | anion transmembrane transporter activity | 0.001417 | 2.848766 | 0.25 |
| GO:0022804 | active transmembrane transporter activity | 0.001776 | 2.750660 | 0.25 |
| GO:0015294 | solute:cation symporter activity | 0.002251 | 2.647659 | 0.166667 |
| GO:0015293 | symporter activity | 0.004014 | 2.396472 | 0.166667 |
| GO:0015081 | sodium ion transmembrane transporter activity | 0.005198 | 2.284172 | 0.166667 |
| GO:0015103 | inorganic anion transmembrane transporter activity | 0.005516 | 2.258350 | 0.166667 |
| GO:0031419 | cobalamin binding | 0.006819 | 2.166271 | 0.083333 |
| GO:0070016 | armadillo repeat domain binding | 0.006819 | 2.166271 | 0.083333 |
| GO:0005215 | transporter activity | 0.008277 | 2.082132 | 0.333333 |
| GO:0051721 | protein phosphatase 2A binding | 0.018986 | 1.721561 | 0.083333 |
| GO:0015075 | ion transmembrane transporter activity | 0.020890 | 1.680068 | 0.25 |
| GO:0015301 | anion:anion antiporter activity | 0.021001 | 1.677765 | 0.083333 |
| GO:0015347 | sodium-independent organic anion transmembrane transporter activity | 0.022342 | 1.650885 | 0.083333 |
| GO:0004712 | protein serine/threonine/tyrosine kinase activity | 0.027688 | 1.557701 | 0.083333 |
| GO:0015077 | monovalent inorganic cation transmembrane transporter activity | 0.028467 | 1.545664 | 0.166667 |
| GO:0022857 | transmembrane transporter activity | 0.032702 | 1.485420 | 0.25 |
| GO:0008146 | sulfotransferase activity | 0.034997 | 1.455975 | 0.083333 |
| GO:0046873 | metal ion transmembrane transporter activity | 0.037584 | 1.424994 | 0.166667 |
| GO:0099516 | ion antiporter activity | 0.040938 | 1.387869 | 0.083333 |
| GO:0016782 | transferase activity, transferring sulfur-containing groups | 0.047501 | 1.323296 | 0.083333 |
