# Supplementary material for: Altered circRNAs: a novel potential mechanism for the functions of extracellular vesicles derived from platelet-rich plasma
Source: Front Bioinform. 2026 Jan 8;5:1690932. doi: 10.3389/fbinf.2025.1690932 (PMC12823818; doi:10.3389/fbinf.2025.1690932)
Supplement: Supplementary file 7 [file Table6.docx]

Supplementary Table 11: The KEGG pathways analysis for the down-regulated circRNAs

| **Pathway ID** | **Definition** | **Fisher P-value** | **Enrichment Score** | **Gene Ratio** |
| --- | --- | --- | --- | --- |
| hsa04964 | Proximal tubule bicarbonate reclamation | 0.000062 | 4.208332 | 0.5 |
| hsa00471 | D-Glutamine and D-glutamate metabolism | 0.002288 | 2.640637 | 0.25 |
| hsa00220 | Arginine biosynthesis | 0.011398 | 1.943159 | 0.25 |
| hsa00532 | Glycosaminoglycan biosynthesis - chondroitin sulfate / dermatan sulfate | 0.011398 | 1.943159 | 0.25 |
| hsa04977 | Vitamin digestion and absorption | 0.013666 | 1.864351 | 0.25 |
| hsa00250 | Alanine, aspartate and glutamate metabolism | 0.019883 | 1.70152 | 0.25 |
| hsa05230 | Central carbon metabolism in cancer | 0.037801 | 1.4225 | 0.25 |
| hsa04976 | Bile secretion | 0.040023 | 1.39769 | 0.25 |
| hsa04727 | GABAergic synapse | 0.049425 | 1.306053 | 0.25 |
| hsa04972 | Pancreatic secretion | 0.053826 | 1.269012 | 0.25 |
| hsa04724 | Glutamatergic synapse | 0.063671 | 1.19606 | 0.25 |
